# Case Report: Neuropsychiatric instability in structurally compromised CNS Erdheim–Chester disease: a diagnostic and management challenge

**DOI:** 10.3389/fpsyt.2026.1752665

**Published:** 2026-04-13

**Authors:** Abdulaziz F. Alfraiji, Mohamed F. Alqahtani, Shahad Aljudi, Fahad D. Alosaimi

**Affiliations:** Department of Psychiatry, College of Medicine, King Saud University, Riyadh, Saudi Arabia

**Keywords:** Erdheim-Chester disease, histiocytosis, neuropsychiatric manifestations, paradoxical drug response, psychotropic sensitivity

## Abstract

**Background:**

Erdheim–Chester disease (ECD) is a rare non-Langerhans cell histiocytosis involving multiple systems, with central nervous system (CNS) involvement present in a significant subset. Neuropsychiatric symptoms such as cognitive decline, mood disturbances, and psychosis have been documented. However, sensitivity to psychotropic medications in CNS-involved ECD remains underreported. This case highlights a multi-mechanistically compromised CNS posing diagnostic and therapeutic challenges by such sensitivity.

**Case presentation:**

A 50-year-old woman with biopsy-confirmed ECD and CNS involvement presented with persistent neuropsychiatric symptom confusion, psychosis, cognitive decline, mood lability, and agitation despite radiologic remission following cladribine and radiotherapy. She exhibited marked sensitivity to multiple psychotropics: haloperidol induced QTc prolongation; risperidone and aripiprazole triggered akathisia and insomnia; olanzapine caused hypotension; chlorpromazine and quetiapine led to oversedation; and benzodiazepines paradoxically worsened agitation. Only valproic acid was tolerated. Factors such as previous chemotherapy, brain radiotherapy, hypernatremia, endocrine dysfunction, and a family history of psychiatric illness were suspected to have a role in predisposition. Imaging showed lesions of the hypothalamus, pituitary stalk, and mesial temporal lobes. Persistent symptoms shared features with Lewy Body Dementia and raised the diagnostic ambiguity.

**Conclusion:**

To our knowledge, this is one of the first detailed reports to describe heightened neuropsychiatric instability and psychotropic intolerance in a patient with a compromised CNS through several suspected mechanisms. The case underscores the need for multidisciplinary care and cautious psychiatric management in patients with a compromised CNS. Clinicians should suspect psychotropic sensitivity in cases with paradoxical responses and consider systemic causes such as ECD as a possible contributor.

## Introduction

Erdheim–Chester disease (ECD) is a rare, multisystem histiocytic neoplasm characterized by the infiltration of foamy, lipid-laden histiocytes in various tissues. While skeletal involvement is nearly universal, ECD can affect almost any organ, with particularly serious consequences when the central nervous system (CNS) is involved (1, 2). Fewer than 1,500 cases have been reported globally, and clinical presentation remains heterogeneous, often leading to delays in diagnosis (1, 3).

CNS manifestations are seen in approximately 30%-40% of patients and may range from cerebellar ataxia to diabetes insipidus and neuropsychiatric symptoms (4). Psychiatric features such as mood changes, anxiety, and cognitive decline are increasingly recognized but remain underreported. These symptoms are often subtle and may precede more typical systemic findings, resulting in misdiagnosis or inappropriate treatment (5, 6).

This case adds to the limited literature on neuropsychiatric involvement in ECD by highlighting a patient who presented primarily with psychiatric symptoms and showed marked sensitivity to psychotropic medications, confounded by a compromised CNS through several hypothesized mechanisms. Psychotropic sensitivity has not been well characterized in ECD and in this case may suggest additional CNS burden in this population.

Our case reinforces the need for clinicians to consider histiocytic disorders in the differential diagnosis of unexplained psychiatric symptoms, especially when accompanied by unusual treatment responses. It also calls attention to the risks of conventional psychotropic prescribing in this context and supports the role of early multidisciplinary assessment and care in complex neuropsychiatric syndromes.

## Case presentation and clinical course

A 50-year-old married female Sudanese living in Saudi Arabia holding a PhD presented with a past medical history of bronchial asthma, type 2 diabetes mellitus, and hypertension. She had no prior psychiatric history. Her family history was notable for bone cancer in an aunt and a cousin, schizophrenia in a brother, and bipolar disorder in an aunt. She was in her usual state of health until January 2020, when she presented to the emergency department with chest pain. Investigations were unremarkable for life-threatening causes. Later that month, she reported progressive bilateral infraorbital swelling. An excisional biopsy with immunohistochemistry (IHC) stains confirmed xanthelasma and xanthogranuloma. Over the following months, she began experiencing recurrent musculoskeletal complains. Serial X-rays revealed hypodensities in the forearms and an opaque tibial lesion. MRI showed a sclerotic lesion, and a bone scan revealed symmetric increased uptake in all limbs, raising suspicion for a metabolic bone disease. Joint aspiration showed an inflammatory profile.

**Figure 1:**
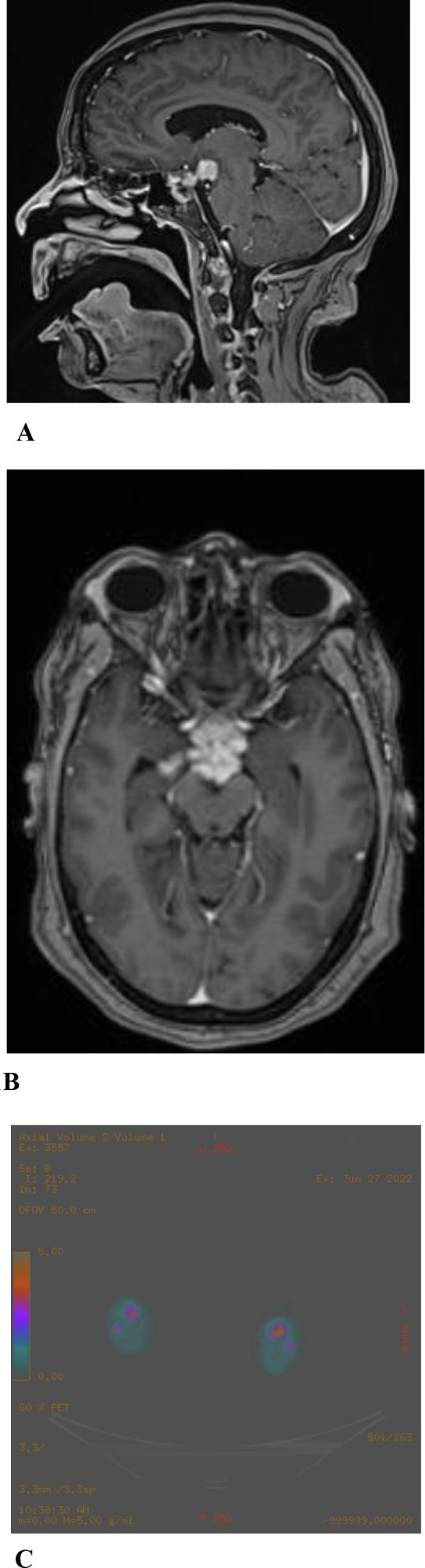
Pre-treatment Radiologic images (2022): Axial **(B)** and sagittal **(A)** post-contrast T1-weighted Brain MRI showing a lobulated enhancing suprasellar mass with infiltration of the pituitary stalk,tuber cinereum and hypothalamus, lamina terminalis and right mesial temporal lobe. Axial **(C)** FDG PET/CT showing bilateral tibial hypermetabolic heterogeneous uptake.

**Figure 2:**
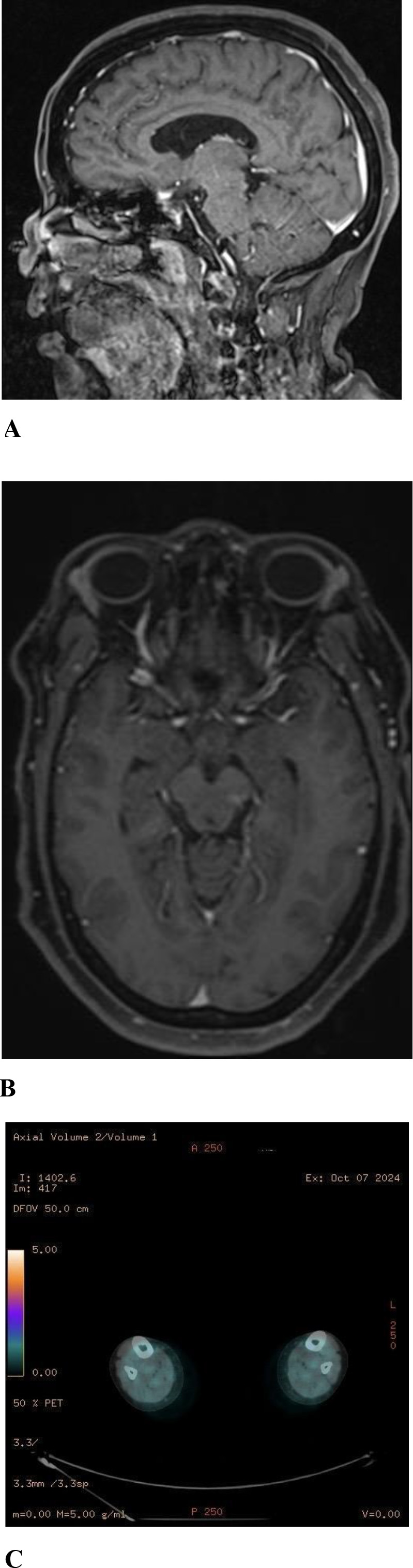
Post-treatment Radiologic images (2024): Axial **(B)** and sagittal **(A)** post-contrast T1-weighted Brain MRI showing complete resolution of the previously seen suprasellar enhancing lesion. No abnormal enhancement seen in the tuber cinereum, hypothalamus, lamina terminalis or interpeduncular cistern. Axial **(C)** FDG PET/CT showing Interval metabolic improvement of the tibial hypermetabolic heterogeneous uptake.

In December 2020, after presenting with stroke-like symptoms and peripheral visual field loss, a CT brain scan incidentally revealed a suprasellar mass. She was also diagnosed with central hypothyroidism, hypogonadism, and long-standing symptomatic hyperprolactinemia. She was started on thyroxine at that time. By early 2022, she began to experience visual hallucinations, delusions, slowed responses, anomia, fluctuating orientation, progressive short-term memory loss, anxiety, loss of interest, daytime sleepiness, reduced appetite, and unintentional weight loss of 11% in a year. A brain MRI showed an enhancing suprasellar mass with involvement of the pituitary stalk, hypothalamus, and right mesial temporal lobe (**Figures 1A, B**). The patient was admitted, and findings raised the suspicion of a histiocytic disorder. By late 2022, an open biopsy of the tibia confirmed a diagnosis of Erdheim–Chester disease (ECD). Four days postoperatively, she became disoriented. After Psychosomatic team involvement, it is believed to be delirium secondary to multiple etiologies. Later that month, she was diagnosed with central diabetes insipidus due to ECD and was started on desmopressin. She tested positive for COVID-19 during a concurrent upper respiratory tract infection. Following recovery, she was empirically started on vemurafenib despite failed BRAF V600E mutation testing twice. However, the disease progressed over the subsequent 2 weeks. This was complicated by an episode of severe hypernatremia (serum sodium (Na) 174 mmol/L, up from a baseline of 145 mmol/L the prior day) which was corrected over 4 days; she also had a prolonged Fridericia-corrected QT interval (QTcFd) of 533 ms from 421 ms, calcium (Ca) (corrected) 2.43 mmol/L, potassium (K) 3.06 mmol/L, and magnesium (Mg) 0.58 mmol/L, leading to discontinuation of vemurafenib. While awaiting cladribine, she received dexamethasone. On the third day of treatment, she developed agitation and aggression, requiring physical and chemical restraint with lorazepam, prompting discontinuation of dexamethasone. She subsequently underwent brain radiation therapy (2,000 cGy in five fractions) without complications, followed by 6-monthly cycles of cladribine, which were well tolerated. Despite some improvements in orientation, mobility, and radiological findings (**Figures 2A–C**), neuropsychiatric symptoms continued to progress. A detailed chronology of the patient’s symptoms and corresponding clinical interventions is summarized in **Table 1**.

**Table 1:** Timeline of symptoms, interventions, and cross-sectional laboratory values.

Timeline	Symptoms/findings	Interventions/investigations	Outcome/notes
Jan 2020	Chest pain	Chest pain workup	No life-threatening cause
Jan 2020	Periorbital swelling	Biopsy showed xanthelasma and xanthogranuloma	Mass excision and blepharoplasty
Feb 2020	Musculoskeletal complains	Bone hypodensity	Bone MRI
Dec 2020	Stroke-like presentation	Brain C.T showed Incidental finding of suprasellar lesion with no stroke findings	Brain MRI
March 2021	Peripheral visual field loss	Labs showed hyperprolactinemia, central hypothyroidism, and central hypogonadism. No adrenal insufficiency	Started on thyroxine Pending brain MRI
April 2021	Bone MRI showed right tibial sclerotic and multifocal lesions	Bone scan showed heterogeneous symmetric increased uptake in bilateral upper and lower limbs	Follow-up for biochemical correlation
Sep 2021	Knee joint pain and swelling	Joint aspiration	Inflammatory picture
Feb 2022	Brain MRI review Stable enhancing suprasellar mass with involvement of the pituitary stalk, hypothalamus and right mesial temporal lobe; ECD is in the differential diagnosis	Bone scan for more evaluation showed increased uptake bilateral tibia	Tibia biopsy
July-Sep 2022	Left tibia biopsy	Pathology report: (Foamy histiocytes, CD138 −ve, pan-CK −ve, CD68 +ve, CD1a −ve) BRAF600E gene mutation testing failed	Diagnosis of ECD
Sep 2022	Disorientation post-op	Surgical site infection was ruled out. Orthopedic surgery’s impression based on clinical assessment: delirium secondary to a medical condition	To treat underlying disease.
19 Oct 2022	Altered level of consciousness	Central diabetes insipidus diagnosis	Admission for desmopressin
20 Oct 2022	Treatment of ECD	Vemurafenib	Discontinued after disease progression and QTcFd prolongation (533 ms from 421 ms, corrected Ca 2.43 mmol/L, K 3.06 mmol/L, and Mg 0.58 mmol/L.)
22 Oct 2022	URTI symptoms	Covid-19 +ve	Azithromycin
25 Oct 2022	Fever	UTI	Ceftriaxone
1 Nov 2022	Vemurafenib is discontinued and cladribine will be given when available	Start bridging with dexamethasone	Discontinued after agitation and aggression then given haloperidol and lorazepam
5 Nov 2022	Disorientation, agitation, reported visual hallucinations, loss of interest, anxiety, and insomnia	Psychosomatic consulted with an impression of delirium (Based on clinical assessment) secondary to a medical condition	Started on valproate and advised to do non-pharmacological interventions for delirium and avoid benzodiazepines. Delirium has been persistent since then
6 Nov 2022	Desmopressin held the day before	Sodium level reached 174 mmol/L from 145 mmol/L the day before	Reintroduce desmopressin
Late Nov 2022	ECD radiotherapy	Brain radiation therapy (2,000 cGy in 5 fractions)	No new complaints
Jan-Feb 2023	One month of psychotropics washout following the discontinuation of haloperidol, the patient reported a decline in sleep quality, the same daily episodes of disorientation to time, place, and person. While there was a noted reduction in hallucinatory gestures and an absence of agitation or disturbed behavior, the patient reported new, sudden episodes of fear accompanied by palpitations, shortness of breath, and tremors, despite no prior clinical history of panic attacks. Labs taken at the end of the month: CBC: WBC (9.13), RBC (4.4), HgB (106), Hct (34.7), MCV (78.7), MCH (24), MCHC (305), RDW (15.4), Plt (246), ANC (7.9), ALC (0.4), AMC (0.7), AEC (0.12), ABC (0.05) WBC: 9.13 × 10^9^/L, RBC: 4.4 × 10¹²/L, hemoglobin (Hgb): 106 g/L, hematocrit (Hct): 34.7%, MCV: 78.7 fL, MCH: 24 pg, MCHC: 305 g/L, RDW: 15.4%, platelets (Plt): 246 × 10^9^/L, ANC (absolute neutrophil count): 7.9 × 10^9^/L, ALC (absolute lymphocyte count): 0.4 × 10^9^/L, AMC (absolute monocyte count): 0.7 × 10^9^/L, AEC (absolute eosinophil count): 0.12 × 10^9^/L, ABC (absolute basophil count): 0.05 × 10^9^/L Other labs (mmol/L): Na (141), K (3.55), Ca (corrected) (2.00-2.37), phosphorus (1.15), Mg (0.72), chloride (106.5), random blood glucose (7.50). Liver and renal panels were normal. Thyroid panel showed subclinical hypothyroidism 1 month before and after this month; TSH (0.349-0.497 mIU/L), free T4 (10.7-8.27 ng/dL).		N/A
Early 2023	ECD chemotherapy	Cladribine 6-monthly cycles	Complete radiological resolution of previously seen brain mass
June 2024	Cognitive decline symptoms	MoCA-A Score 14	Follow-up
Jan 2025	Cognitive decline symptoms	MoCA-A Score 14	Follow-up
April 2025	Cognitive decline symptoms	MoCA-B Score 18	Follow-up

MoCA-B, Montreal Cognitive Assessment Basic.

ECD, Erdheim–Chester Disease.

QTcFd, Fridericia-corrected QT interval.

Initially, following the 3 days of dexamethasone administration leading to agitation and aggression under the impression of hyperactive delirium due to multiple etiologies, she received lorazepam and haloperidol (2 mg each, given twice) but remained agitated and had a prolonged QTc (515 ms, from a baseline of 456 ms 5 days prior, with Ca (corrected) 2.64 mmol/L, K 3.43 mmol/L, and unknown Mg). She then received a single 250-mg dose of valproic acid, and the primary team was advised to use non-pharmacological delirium interventions and avoid deliriogenic medications. Valproic acid, dosed at 250 mg twice daily with PRN options, yielded partial benefit for agitation and sleep. It emerged as the most sustainable agent, despite the development of hand tremors after 2 years of long-term use.

Psychotic symptoms persisted, prompting the addition of aripiprazole, titrated to 7.5 mg once daily, which did not improve symptoms and caused akathisia (evaluated by a clinical assessment) and insomnia; the latter did not improve with a course of melatonin 10 mg HS. Valproic acid was discontinued, and haloperidol was cross tapered with aripiprazole and titrated to 2.5 mg once daily, reducing some of the agitation and psychotic symptoms. Aripiprazole was eventually discontinued, and haloperidol was increased to 5 mg once daily, although higher doses led to oversedation and constipation leading to a dose reduction. The patient was discharged on haloperidol 2.5 mg once daily. As part of the psychosomatic out-patient out-reach services, she was contacted after discharge and instructed to discontinue haloperidol due to concerns about QTcFd prolongation since the last reading was 495 ms, from a baseline of 464 ms, with calcium (Ca) (corrected) 2.33 mmol/L, potassium 3.74 mmol/L, and magnesium 0.72 mmol/L. A month later, on her next admission, she remained psychotic and was restarted on haloperidol 1.5 mg once daily, but slight QTcFd prolongation recurred (484 ms from a baseline of 434 ms, Ca (corrected) 2.25 mmol/L, K 3.57 mmol/L, and Mg 0.77 mmol/L), leading to discontinuation and initiation of olanzapine, titrated to 2.5 mg HS. Olanzapine improved sleep and psychosis but caused oversedation and hypotension; at a reduced dose of 1.25 mg once daily, oversedation and psychosis persisted with intermittent agitation, leading to discontinuation eventually. Valproic acid 250 mg twice a day was restarted, improving agitation but not psychosis, so risperidone 0.25 mg HS was added but caused akathisia (based on clinical assessment) and insomnia prompting its discontinuation. A MoCA-A performed at that time was 14. Escitalopram was started and titrated to 10 mg once daily for anxiety with no notable outcome. Quetiapine was initiated and titrated to 25 mg, improving sleep and partially improving psychosis, but at 100 mg, the patient became irritable. During this period, the valproic acid level was 29.1 mg/L and the patient was admitted to the ICU for viral pneumonia. Quetiapine was then switched to chlorpromazine 25 mg once daily, which did not improve psychosis; at 50 mg once daily, the patient developed oversedation.

As of September 2025, there is no documented history of REM sleep behavior. Her current psychotropics are valproic acid 250 mg twice daily, escitalopram 10 mg once daily, and chlorpromazine 25 mg once daily. She remains psychotic but not aggressive and reports worsening cognitive functioning. She was referred to Geriatric Psychiatry for further neuropsychiatric evaluation with an impression of mixed type of neurocognitive disorders (Alzheimer with Lewy Body disease), although there is still a possibility of episodes of delirium due to the underlying medical conditions. Furthermore, she was referred to Neurology’s Neurocognitive clinic, where it was concluded that neurodegenerative diseases were less likely given the absence of tau and amyloid proteins and normal CSF composition—documented during a Neurology outpatient visit based on external laboratory results. Although not definitive, further evaluation with brain FDG-PET did not demonstrate a characteristic pattern of hyper- or hypometabolism suggestive of a specific neurodegenerative disease entity. All documented and prescribed psychotropics are summarized in **Table 2** with respective dose range, duration, therapeutic effect, adverse reactions, and current status.

**Table 2:** Medications and their effects.

Medication	Dose range	Longest trial duration	Purpose	Favorable effect	Adverse events/notes	Status
Valproic acid	250 mg twice daily with as-needed options	+2 years	Psychosis and agitation	Partially improved agitation and sleep	Hand tremors	Current, serum level 29.1 mg/L (measured once)
Haloperidol	1.0–5.0 mg	6 weeks	Agitation	Reduced agitation and psychosis	Oversedation (BP 100-147/51–88 mmHg), constipation, and QTcFd prolongation (467 ms, baseline of 424 ms, with corrected Ca 2.33 mmol/L, K 3.74 mmol/L, and Mg 0.72 mmol/L.)	Discontinued
Olanzapine	2.5 mg	7 weeks	Psychosis	None	Oversedation and hypotension (BP 140-149/87–47 mmHg)	Discontinued
Aripiprazole	2.5–7.5 mg	5 weeks	Psychosis	None	Akathisia and insomnia	Discontinued
Risperidone	0.25 mg	14 weeks	Psychosis	None	Akathisia and insomnia	Discontinued
Quetiapine	12.5–100 mg	11 weeks	Psychosis and sleep	Improved sleep and psychosis	Higher doses associated with increased irritability ICU admission due to pneumonia	Discontinued
Chlorpromazine	25–50 mg	5 weeks	Psychosis	None	Oversedation (BP 139-152/74–84 mmHg)	Current
Escitalopram	10 mg	16 weeks	Anxiety	None reported	None	Current
Midazolam	0.5 mg	As-needed	Agitation	None	Increased agitation	Discontinued
Lorazepam	2 mg	As-needed	Agitation	None	Increased agitation	Discontinued
Melatonin	5–10 mg	Unknown	Sleep	None	None	Discontinued

## Discussion

Neuropsychiatric symptoms, including depression, anxiety, cognitive impairment, and personality changes, are increasingly acknowledged in ECD, with CNS involvement seen in up to 51% of patients (3, 6–8). However, psychosis remains uncommon, and sustained psychiatric symptoms following systemic disease control have been only rarely described in the literature (6). This case contributes to the limited body of evidence, suggesting that long-term neuropsychiatric sequelae may persist in CNS-ECD even after apparent radiologic or systemic stabilization. In contrast, one study reported rapid resolution of psychosis in a BRAFV600E-mutated ECD patient treated with vemurafenib and interferon-α (9), whereas another described improvement in affective symptoms following systemic therapy (6).

The persistence of psychiatric symptoms in our patient despite radiologic remission and systemic disease control raises the possibility that residual or secondary CNS dysfunction, rather than ongoing active histiocytic infiltration, may underlie the clinical course. To conceptualize this, we propose a theoretical “multi-hit” model of CNS vulnerability, in which cumulative structural and systemic insults may interact within an already compromised neural substrate. Potential contributors include chemotherapy-related cognitive effects (10), radiation-induced inflammatory and microvascular brain changes (11), severe hypernatremia–associated neurologic stress (12), and SARS-CoV-2–related neuroinflammatory mechanisms (13). Although this framework remains hypothetical, such cumulative insults have been independently associated with persistent alterations in neuronal, glial, and endothelial function, which may plausibly lower the threshold for enduring neuropsychiatric dysfunction.

The reproducible cross-class intolerance suggests heightened central pharmacodynamic sensitivity rather than isolated drug-specific reactions. Paradoxical agitation with benzodiazepines has been described in susceptible individuals (14), and delirium itself may present with psychotic features and fluctuating behavioral instability in medically complex patients (15). In this context, the relative tolerability of valproic acid, an agent that enhances GABAergic tone and stabilizes neuronal excitability (16), may indicate that modulation of inhibitory pathways was better tolerated than dopaminergic antagonism. Valproic acid has also been investigated as a therapeutic option for hyperactive delirium and agitation in critically ill patients (17), supporting its potential role in stabilizing behavioral dysregulation in vulnerable CNS states. Moreover, given the established role of dopamine receptor blockade in extrapyramidal and behavioral adverse effects (18), structural disruption of limbic–hypothalamic circuits may have lowered the threshold for such reactions. Direct histiocytic infiltration of the hypothalamus and mesial temporal lobes regions integral to dopaminergic, limbic, and autonomic regulation may have altered network-level modulation of motor and affective circuits (4, 6, 19). Injury to frontal and limbic networks has been associated with behavioral disinhibition and exaggerated responses to centrally acting medications (15). Collectively, these structural vulnerabilities may have contributed to altered pharmacodynamic responsiveness in this patient.

Beyond structural network vulnerability, hypothalamic involvement in this case raises the possibility that persistent neuroendocrine dysregulation contributed to affective and behavioral instability. The hypothalamus functions as a central integrator of endocrine and autonomic stress responses, coordinating hypothalamic–pituitary axes while interfacing with limbic circuits involved in mood regulation (14). Disruption of this regulatory hub may therefore produce enduring alterations in stress responsivity even without radiologic progression. The patient’s central hypothyroidism, diabetes insipidus, and hyperprolactinemia reflect significant hypothalamic–pituitary axis dysfunction. Thyroid hormones are critical for cerebral metabolism and neurotransmitter regulation, and disturbances in thyroid signaling have been associated with depressive symptoms, cognitive impairment, and mood instability (16). In addition, hypothalamic injury may alter regulation of the HPA axis, potentially contributing to affective lability and impaired stress tolerance (14).

Cardiac involvement is common in ECD and may further complicate psychotropic management. Pericardial effusion, myocardial infiltration, and conduction abnormalities have been reported in this population (1, 2). Antipsychotic medications are known to prolong the QTc interval and, in susceptible individuals, may increase the risk of torsades de pointes and other ventricular arrhythmias (17). In this context, underlying cardiac involvement may further increase vulnerability to arrhythmogenic risk.

In addition to structural and endocrine mechanisms, several secondary neurologic stressors may have compounded CNS vulnerability in this patient. Chemotherapy-related neurotoxicity and radiation-induced inflammatory and microvascular changes have been associated with persistent neuronal and glial alterations (10, 11). A documented episode of severe hypernatremia (serum sodium 174 mmol/L) also represents a significant neurologic stressor. Severe sodium derangements and their correction have been associated with osmotic shifts and structural brain injury, including demyelination, depending on chronicity and rate of correction (12). Emerging evidence suggests that SARS-CoV-2 infection may trigger neuroinflammatory responses through endothelial and microglial activation, potentially affecting blood–brain barrier integrity and central immune signaling (13). COVID-19–associated sodium disturbances have also been described and may further compound neurologic vulnerability in susceptible individuals (20). Although none of these factors can be definitively implicated as causal, their cumulative impact is hypothesized to contribute to persistent neuropsychiatric dysfunction in a structurally vulnerable CNS.

Radiotherapy to hypothalamic and suprasellar regions is known to induce delayed inflammatory and microvascular changes (11). Experimental and clinical studies have described radiation-associated endothelial dysfunction and altered vascular permeability in irradiated brain tissue. While direct compromise of the blood–brain barrier (BBB) or altered psychotropic pharmacokinetics cannot be established in this case, regional irradiation is hypothesized to theoretically have an increased local vulnerability within already infiltrated limbic–hypothalamic circuits. Subclinical hepatic and renal involvement, both reported in ECD (1, 2), could theoretically influence psychotropic metabolism and clearance.

Clinically, fluctuating cognition, visual hallucinations, and neuroleptic sensitivity raised suspicion for Lewy Body Dementia (LBD) (21). However, early age of onset (50 years old), lack of REM sleep behavior by history, non-medication-induced parkinsonism, presence of hypothalamic and mesial temporal lobe lesions, suprasellar mass, absence of a definitive pattern of a neurodegenerative disease in brain FDG-PET, absence of CSF tau and amyloid proteins, endocrine dysfunction, and electrolyte derangements provide a more comprehensive explanation of this complicated case. Still, this overlap illustrates the diagnostic ambiguity of neuropsychiatric symptoms in a rare systemic disease superimposing on a compromised CNS. Also, an additional complexity is the family history of schizophrenia and bipolar disorder, suggesting genetic vulnerability. While no direct causal relationship can be established, genetic vulnerability may influence symptom expression when structural or metabolic CNS compromise is present.

Cardiac involvement is common in ECD and may further complicate psychotropic management. Pericardial effusion, myocardial infiltration, and conduction abnormalities have been reported in this population (1, 2). Antipsychotic medications are associated with QTc prolongation and arrhythmogenic risk, including torsades de pointes, particularly in medically complex or critically ill patients treated for delirium (22, 23). Given this established pharmacologic effect, underlying cardiac involvement may further increase vulnerability to arrhythmias and necessitate careful ECG monitoring. Additionally, clinicians must therefore adopt an individualized, cautious prescribing strategy, including low-dose initiation, gradual titration, preference for lower extrapyramidal-liability agents such as quetiapine, and vigilant ECG monitoring (21, 22). Interestingly, valproic acid, an agent with GABAergic and anti-kindling properties (16), showed partial efficacy and was best tolerated. This pharmacologic contrast may suggest that targeting inhibitory stabilization rather than dopaminergic blockade is better tolerated in structurally vulnerable networks.

Non-pharmacologic strategies, including environmental stability and structured routines, played a pivotal role. Interdisciplinary collaboration was essential to minimize iatrogenic harm and optimize care. Future research into the neurochemical mechanisms of CNS-ECD and psychotropic response could inform more tailored therapeutic approaches.

## Conclusion

This case underscores a rare and diagnostically challenging presentation of Erdheim–Chester disease complicated by a compromised CNS through several suspected additional mechanisms, leading to heightened neuropsychiatric instability, profound psychotropic sensitivity, and limited pharmacologic options despite systemic remission. The observed intolerance to multiple medication classes highlights the importance of recognizing CNS vulnerability in such cases with careful anticipation of iatrogenic risks.

To our knowledge, this is among the first detailed reports documenting multiagent psychotropic intolerance in ECD in a compromised CNS through several confounding suspected mechanisms. Clinicians managing such patients must proceed with heightened caution, guided by comprehensive cardiac, neurologic, and endocrine evaluation, careful low-dose regimens, and non-pharmacologic strategy prioritization. Enhanced clinical vigilance and interdisciplinary collaboration are critical for improving diagnostic precision and therapeutic outcomes. Further research is needed to clarify mechanisms driving CNS involvement and the suspected predisposing role of histiocytic disorders in psychotropic hypersensitivity given the limitations of the complexity of multiple confounding CNS insults in this case.

## Data Availability

The raw data supporting the conclusions of this article will be made available by the authors, without undue reservation.
